# Meesmann Corneal Dystrophy with Epithelial Basement Membrane Abnormalities: Clinical and Genetic Analysis of Two Families with Novel and Known Mutations in *KRT3* and *KRT12*

**DOI:** 10.3390/ijms27031326

**Published:** 2026-01-29

**Authors:** Víctor Charoenrook, Raquel Larena, Álvaro Ferragut-Alegre, Alix De Faria, Rebeca Valero, Mònica Martí-Orpinell, Gemma Julio, Rafael I. Barraquer

**Affiliations:** 1 Centro de Oftalmología Barraquer, 08021 Barcelona, Spain; charoenrook@barraquer.com (V.C.); raquel.larena@barraquer.com (R.L.); doctorferragut@gmail.com (Á.F.-A.); monica.marti@barraquer.com (M.M.-O.);; 2 Institut Universitari Barraquer, Universitat Autònoma de Barcelona, 08021 Barcelona, Spain; 3 DBGen Ocular Genomics, 08028 Barcelona, Spain; 4 School of Medicine, Universitat Internacional de Catalunya, 08017 Barcelona, Spain

**Keywords:** Meesmann epithelial corneal dystrophy, *KRT3*, *KRT12*, in vivo confocal microscopy, anterior segment optical coherence tomography, epithelial basement membrane dystrophy

## Abstract

This study describes the clinical and genetic features of Meesmann epithelial corneal dystrophy (MECD) in two unrelated families and reports new genotype–phenotype associations. Ten patients from a Lebanese family (*n* = 4) (Family 1) and a Spanish family (*n* = 6) (Family 2) underwent ophthalmologic evaluation, in vivo confocal microscopy (IVCM), anterior segment optical coherence tomography (AS-OCT) with epithelial thickness mapping (ET-map), and targeted next-generation sequencing (NGS) using a custom-designed 133-gene panel associated with anterior segment dystrophies. In Family 1, a novel homozygous *KRT12* c.1181T>C (p.Leu394Pro) variant was identified in the symptomatic proband and his clinically asymptomatic brother, while both parents, who were first cousins, were heterozygous for this nucleotide variant. The proband also carried the heterozygous *KRT3* c.250C>T (p.Arg84Trp) variant, which has been previously reported but, to our knowledge, has not been described in co-occurrence until now. In addition, the proband showed a complex phenotype with signs of MECD and epithelial basal membrane alterations consistent with epithelial basement membrane dystrophy (EBMD). In Family 2, four affected members carried the *KRT3* c.1492G>A (p.Glu498Lys) variant in heterozygosity, which has been previously described. The elderly members affected showed typical signs of MECD and EBMD. To our knowledge, these concomitant alterations have not been previously described with genetical confirmation. In conclusion, this study provides the first evidence that the co-occurrence of variants in two Meesmann corneal dystrophy-associated genes *(KRT3* and *KRT12*) can jointly account for the disease phenotype. We also highlight the association of MECD with EBMD in both families. Characterization using IVCM and AS-OCT ET-Map provides a deeper understanding of the morphological changes and phenotypic variability in MECD, confirming the utility of this multimodal imaging approach for diagnosis and management.

## 1. Introduction

Meesmann epithelial corneal dystrophy (MECD) is an autosomal dominant disorder with incomplete penetrance due to mutations in keratin production genes, that is, *KRT3* and *KRT12* [1,2,3]. These genes encode type II and type I keratins, respectively, which are essential for the differentiation processes and mechanical integrity of corneal epithelial tissues. Mutations in these genes lead to mechanical fragility of the corneal epithelium [4].

There is no racial or ethnic origin that is most commonly affected. Cases have been described in Europe, Asia, and America. Currently, the global prevalence of affected cases is unknown because there is no global database. Therefore, novel mutation identification is necessary to avoid misdiagnosis and to improve clinical management and treatment approaches.

In MECD patients, the corneal epithelium is characterized by the presence of small, rounded epithelial cysts, which may increase with age, and multiple grey punctate micro-opacities distributed in the interpalpebral zone and extending to the limbus of the cornea, visible by biomicroscopy under direct or retroillumination [2,5].

Although MECD is often asymptomatic, the rupture of the tiny cysts causes symptoms such as photophobia, lacrimation, foreign body sensation, corneal erosion, and blurred vision [1,2,3].

Most symptomatic cases are treated with topical lubricant drops or ointments, cycloplegia, and therapeutic soft contact lenses [6], but those with severe opacities and visual impairment may require management with epithelial debridement [7], phototherapeutic keratectomy [8,9], lamellar or penetrating keratoplasty [10], or other recent therapeutic approaches, such as Immunosafe Plasma in Growth Factors eyedrops [11,12]. Unfortunately, MECD may recur after these treatments.

Diverse gene-based therapies have been proven in a human MECD cell line to reduce mutant K12 expression and formation of aggregates, such as allele-specific small interfering RNA (iRNA), with propitious results [13]. Similarly, CRISPR/Cas9 showed favorable results in inhibiting mutant K12 expression in vivo [14], but the efficacy and safety of these emerging therapies for clinical use require further analysis [15].

Anterior segment optical coherence tomography (AS-OCT) and in vivo confocal microscopy (IVCM) are non-invasive techniques that help clinicians in the characterization and diagnosis of the disease, providing data about epithelial thickness and cellular structures changes caused by corneal dystrophies [16].

We present a clinical and genetic study of two families with MECD. The first is a Lebanese family in which we identified the coexistence of a novel, likely pathogenic, nucleotide variant *KRT12* gene (c.1181T>C, p.Leu394Pro), together with a previously described mutation in the *KRT3* gene (c.250C>T, p.Arg84Trp) [17].

The second family is from Spain and is affected by a previously described KRT3 mutation (c.1492G>A) [18]. The elderly affected members of this family showed not only epithelial alterations but also epithelial basement membrane dystrophy (EBMD).

The aim of this study is to provide a comprehensive description of the clinical and molecular findings of the disease in these families using IVCM and AS-OCT, enhancing the understanding of the phenotypic variability and clinical management of MECD.

## 2. Results

### 2.1. Clinical Findings and Complementary Tests

In Family 1, the proband (II-2), a 30-year-old male, had an ocular history during childhood, with episodes of incapacitating pain, photophobia, foreign body sensation, decreased visual acuity (VA), and recurrent corneal erosions interspersed with asymptomatic periods. A year before, the patient visited another ophthalmologic center, and he was treated with standard bandage soft lenses, but the keratopathy always recurred, and scleral lenses were suggested as an alternative treatment. In the other center, a central corneal biopsy (OD) (0.5 × 0.5 × 0.2 mm) of hazy membranous tissue without pigmentation was also performed, with no evidence of microorganisms, including microsporidia. A neoplastic nature of the lesions was disproven by toluidine blue stain.

**Figure 1:**
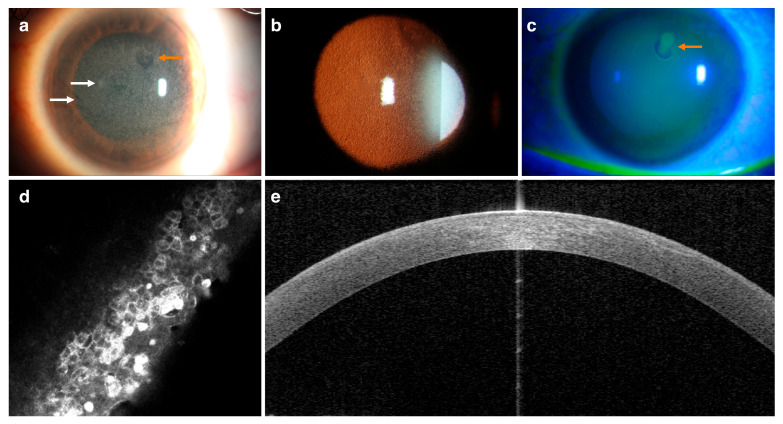
Images of the left eye of the proband in Family 1 (patient II-2). Corneal slit-lamp biomicroscopy (**a**) and retroillumination images (**b**) display multiple dense intraepithelial microcysts and fingerprint lines (white arrows) in the corneal epithelium. Fluorescein vital (**c**) staining is evidence of an aberrant epithelium in the superior temporal area (orange arrows). IVCM (**d**) illustrates multiple microcysts and hyperreflective material throughout the corneal epithelium (IVCM images were acquired covering a 400 × 400 µm area with lateral resolution 1 µm/pixel). AS-OCT is evidence of an irregular and thickened epithelial basement membrane insinuating into the corneal epithelium (**e**).

The onset slit-lamp examination in our center revealed multiple tiny bilateral diffuse intraepithelial cysts, predominantly condensed in the central area of the epithelium and extending to the cornea’s limbus, and map-dot-like lesions (see Figure 1). The OD also showed a 6 mm round area of elevated epithelium due to the previous epithelial biopsy. The left eye (OS) presented a small round area of aberrant epithelium in the superior part of the cornea. The ocular treatment for both eyes approach was eyelid wipes (Naviblef^®^; Novax Pharma, Monaco) two times per day, hypertonic drops (every 8 h) and ointment (at night) (ODM 5^®^, Horus Pharma, Saint-Laurent-du-Var, Niza), and artificial tears every 2–3 h (Hylo Comod^®^; Ursapharm Arzneimittel GmbH, Saabrücken, Germany) to relieve irritation and reduce inflammation.

Fifteen days after the first consultation, the patient presented with an episode of acute pain, lacrimation, blurred vision, and photophobia lasting 5 days in the OS. Ophthalmological evaluation revealed an increased density of intraepithelial cysts with epithelial erosion and visual loss in the OS (0.70 LogMAR). After 2 months of the same treatment, the episode subsided, with VA recovering (0.0 LogMAR), and the patient was asymptomatic one week after the onset of symptoms.

The slit-lamp biomicroscopy examination on patient I-2 (proband’s mother) revealed few isolated intraepithelial cysts with no symptoms. The father (I-1) and sibling (II-2) were asymptomatic, with no evidence of microcysts or alterations on slit-lamp examination. See Table 1 for the demographic and clinical characteristics of each family member.

Regarding the esthesiometry and Schirmer I test, all patients showed normal values. Only the non-invasive tear breakup time (NIBUT) was altered in patient II-1 (OD: 9.1 s and OS: 2.8 s), and the proband (II-2) showed altered values only in the right eye (1.1 s), in which the biopsy had been performed.

**Figure 2:**
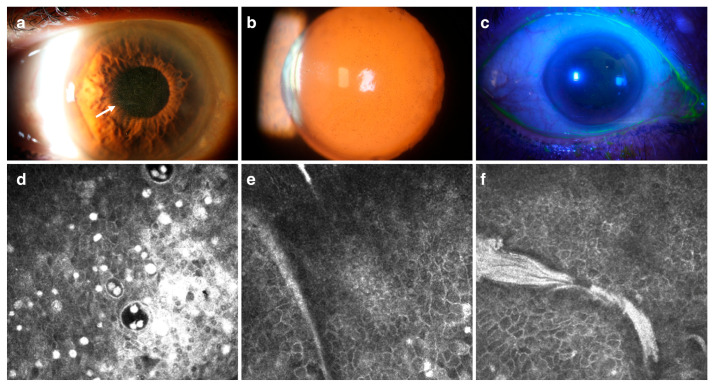
Image of the right eye of the proband in Family 2 (patient I-2). Slit-lamp (**a**), retroillumination (**b**), and fluorescein with vital stain (**c**) reveal multiple intraepithelial microcysts and map-dot–fingerprint dystrophy (white arrow). IVCM shows hyperreflective material and intraepithelial microcysts with well-defined edges in the epithelium (**d**); basal folds correspond to abnormal basement membrane deposits within basal epithelial cells (**e**,**f**) (IVCM images were acquired covering a 400 × 400 µm area, resulting in a lateral resolution of 1 µm/pixel).

In Family 2, ophthalmic evaluation revealed multiple corneal intraepithelial microcysts and whitish opacities in the proband (I-2), a 72-year-old female, and two affected adult patients (II-1) and (II-3). Subepithelial scars were present in the proband and in the patient (II-1). In addition, the proband (I-2) and patient (II-1) revealed map-dot–fingerprint lesions as a manifestation of EBMD (see Figure 2). The affected child patient (III-1) exhibited diffuse epithelial punctate lesions without the presence of microcysts. The two unaffected patients, (II-2) and (III-2), had no visible corneal alterations. Dry eye tests were within normal values for all individuals, except for unaffected patient II-2, who had an altered Schirmer’s test (7mm OD and 5mm OS), probably related to his job as a carpenter (see Table 1).

### 2.2. Genetic Findings

#### 2.2.1. Genetic Analysis of Family 1

**Figure 3:**
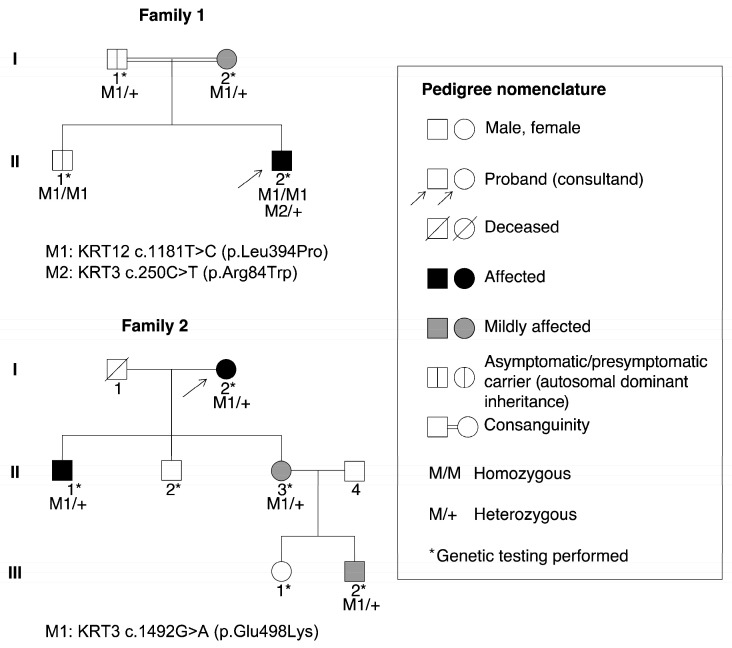
Pedigrees of Families 1 and 2.

Next-generation sequencing (NGS) testing revealed that the proband (II-2) was homozygous for *KRT12* (NM_000223.4) c.1181T>C (p.Leu394Pro) and heterozygous for the *KRT3* c.250C>T (p.Arg84Trp) variant (NM_057088.3). Co-segregation analysis by Sanger sequencing confirmed the proband’s genotype and showed that both parents were heterozygous carriers of *KRT12* c.1181T>C (p.Leu394Pro). Furthermore, this analysis revealed that the asymptomatic sibling (II-1) was also homozygous for *KRT12* c.1181T>C (p.Leu394Pro). Co-segregation analysis of the *KRT3* c.250C>T (p.Arg84Trp) variant could not be performed (see pedigree in Figure 3).

The *KRT12* c.1181T>C variant results in a leucine-to-proline substitution at residue 394. This variant was classified as likely pathogenic according to the American College of Medical Genetics and Genomics (ACMG) guidelines. Multiple pathogenicity prediction algorithms support a deleterious effect, and its low population frequency (f = 0.0000442 in gnomAD) is consistent with potential pathogenicity. Additionally, residue 394 is highly conserved across species. Structural analysis using the HOPE server developed at the Centre for Molecular and Biomolecular Informatics at Radboud University in Nijmegen [19], indicates that the p.Leu394Pro substitution introduces an amino acid with different physicochemical properties, which may disrupt the protein domain. The affected residue, Leu394, is located within a region annotated in UniProt as “Coil 2” and is predicted by Reprof software (http://rostlab.org) to reside in an alpha-helix. Consequently, the introduction of a proline residue might disrupt the alpha-helix structure, potentially causing severe functional effects. Regarding the *KRT3* c.250C>T (p.Arg84Trp) variant, found in heterozygosity in the proband, it has been previously reported as a variant of uncertain significance in an individual with MECD [17]. However, ClinVar classifies it as benign according to ACMG guidelines. Although co-segregation analysis was not possible for this specific variant (*KRT3* c.250C>T (p.Arg84Trp)), the genetic results suggest a potential additive pathogenic effect of both variants in Family 1.

#### 2.2.2. Genetic Analysis of Family 2

NGS panel testing, validated by Sanger sequencing, identified the heterozygous *KRT3* (NM_057088.3) missense variant c.1492G>A (p.Glu498Lys) in all affected members of Family 2 (I-2, II-1, II-3, and III-2) (see pedigree in Figure 3). This variant was absent in unaffected relatives (II-2 and III-1). The c.1492G>A substitution results in a p.Glu498Lys amino acid change in the *KRT3* protein and has been previously reported as pathogenic in MECD associated with pseudo-unilateral lattice corneal dystrophy in another Spanish family [18]. The *KRT3* genotype observed in Family 2 is consistent with an autosomal dominant inheritance pattern, and concordant evidence from previous studies further supports the pathogenicity of *KRT3* c.1492G>A (p.Glu498Lys) in this family.

### 2.3. In Vivo Confocal Microscopy (IVCM) Findings

In Family 1, IVCM images for the proband (II-2) revealed numerous intraepithelial pleiomorphic cysts with well-defined edges and multiple hyperreflective materials (cellular debris) within and outside the microcysts. The microcysts’ diameter ranged from 15.6 to 55.3 µm, with a density of 98.4 ± 4 (OD) and 106 ± 9 microcyst/mm^2^. The surface cells were flattened and degenerated. The basal epithelial cells displayed numerous hyperreflective materials and visible nuclei, along with extracellular deposits. In addition to the presence of tortuous subepithelial nerves, there was atrophy of Bowman’s layer with active keratocytes and highly reflective needle-shape structures in the stroma (see Table 2 and Figure 1).

In Family 2, IVCM confirmed the presence of numerous intraepithelial microcysts with well-defined edges in the proband (I-2). The microcyst density was 25 ± 2 (OD) and 34 ± 4 (OS) cysts/mm^2^, and the diameter ranged from 58.3 to 15.6 µm. Hyperreflective material, corresponding to cellular debris, was present both inside and outside the microcysts, with a density of 261 ± 14 cellular debris/mm^2^. Microcysts and hyperreflective material were located at depths between 3 µm and 55 µm. The images further showed an abnormal basement membrane with folds, Bowman’s membrane atrophy (compatible with EBMD), subepithelial nerve abnormalities, and active keratocytes with close keratocyte–nerve interaction in the stroma (see Table 2 and Figure 2).

The affected relatives (II-1 and II-3) showed similar corneal epithelial abnormalities of MECD on biomicroscopy, AS-OCT, and IVCM (see Table 2 for IVCM).

### 2.4. Epithelial Thickness Map (ET-Map) and Anterior Segment Optical Coherence Tomography (AS-OCT)

In Family 1, the proband (II-2) displayed diffuse hyperreflectivity in the corneal epithelium and Bowman’s layer on AS-OCT. The corneal epithelial layer evidenced an irregular epithelial basement membrane with duplication associated with undulation and elevation. The ET-map showed a slight thickening in the central (OS: mean of 52 µm; range 47–54 µm), inferonasal, and interpalpebral areas. The optical coherence tomography (OCT) image in Figure 1 shows the basal membrane alterations.

The proband’s mother showed a similar pattern with an increased interpalpebral zone mean central thickness of 56 µm, range: 52–60 µm (OD)/50 µm; range: 48–52 µm (OS).

In Family 2, AS-OCT images revealed diffuse hyperreflectivity in the corneal epithelium and Bowman’s layer in the three affected patients (3/6). The proband (I-2) revealed the presence of an irregular and thickened epithelial basement membrane insinuating into the corneal epithelial layer, related to EBMD. In affected patients (I-2, II-1, II-3), ET-map revealed a thicker corneal epithelium in the central and inferior regions corresponding to the visual axis and midperiphery area, with a thinning superior epithelium. The central epithelial thickness in affected patients ranged from 65 µm to 79 µm in OD (mean: 70.07 ± 7.57 µm) and 62 µm to 82 in OS (mean: 69.84 ± 10.41 µm). Child patients (III-1, III-2) did not undergo AS-OCT.

## 3. Discussion

Acidic keratins (K9–K21) and neutral–basic keratins (K1–K8) are crucial components of all epithelial membranes. A pair of keratins forms a tissue-specific heterodimer; disruption of either keratin within this heterodimer compromises the integrity of the epithelial cell layer [20]. K3 and K12 are essential components of the intermediate filament cytoskeleton of the corneal epithelium, and mutations within either *KRT12* or *KRT3* are known causes of MECD.

This study provides a detailed clinical, imaging, and molecular analysis of two families with MECD associated with EBMD. In the Lebanese family (Family 1), we report a novel, disease-causing variant in *KRT12* as the main genetic cause of the disease, together with a previously described variant in *KRT3*. The available evidence does not support a true digenic inheritance mechanism; instead, our data are consistent with a model in which the *KRT3* variant acts as a genetic modifier influencing the phenotypic expression of the novel *KRT12* mutation.

This study provides a detailed clinical, imaging, and molecular analysis of two families with MECD associated with EBMD while also identifying a novel digenic finding in a Lebanese family (Family 1) with a complex phenotype.

Indeed, in Family 1, we report for the first time a case of MECD in whom disease-causing mutations in the *KRT12* and *KRT3* genes coexist.

Specifically, the proband (II-2) was homozygous for a novel missense variant in *KRT12* (c.1181T>C, p.Leu394Pro), classified as likely pathogenic according to ACMG criteria (class 4), and was also heterozygous for a previously described *KRT3* variant (c.250C>T, p.Arg84Trp) [17]. The genetic landscape appears more complex in this case, and the disease was more severe (early onset, more visual impairment, with several painful episodes of recurrent corneal erosions). The results suggest that the *KRT12* c.1181T>C (p.Leu394Pro) variant is the primary driver of the disease. Structural modeling supports this, as the mutation occurs in a highly conserved region and introduces a proline into an alpha-helix, a change classically associated with helix disruption and structural instability. However, the phenotype in the proband (II-2) appears more severe than expected, potentially due to the presence of the *KRT3* (c.250C>T, p.Arg84Trp) variant in heterozygosity. While *KRT3* c.250C>T has been classified as benign in some databases, its co-occurrence with *KRT12* (c.1181T>C, p.Leu394Pro) suggests a modifying effect, contributing to the severity of the phenotype. It is worth pointing out that this variant has been previously reported as a variant of uncertain significance in *KRT3* in an individual with MECD [17].

This hypothesis of accumulative disease-causing effect is plausible, particularly given the consanguinity in the Family 1 progenitors, which explains the *KRT12* homozygosity. Notably, although mutation hot spots in keratin genes are typically found within the helix-initiation and helix-termination motifs, previous studies have demonstrated that variants altering residues outside these regions can also cause MECD. Therefore, we propose that *KRT12* c.1181T>C establishes the primary disease-causing background in Family 1, whereas *KRT3* c.250C>T may act as a genetic modifier contributing to the observed clinical variability.

Regarding genetic analysis of Family 2, the identification in heterozygosity of the *KRT3* missense variant (c.1492G>A, p.Glu498Lys) in all affected members reinforces the pathogenicity of this nucleotide variant. The p.Glu498Lys mutation affects a strongly conserved residue within the 2B subdomain of the intermediate filament chain. This finding aligns with previous reports linking this variant to MECD phenotypes. This specific mutation has been reported only once before [18], also in a Spanish patient, who presented with MECD coexisting with pseudo-unilateral lattice corneal dystrophy. Notably, this mutation, along with the c.1492A>T mutation described by Szaflik et al. (2008) [21], codes for the same amino acid.

A significant finding in both families is the coexistence of MECD with other distinct corneal abnormalities. The proband (II-2) in Family 1 and the proband (I-2) and patient (II-1) in Family 2 presented with signs of EBMD. However, this association is not absolute; another affected but asymptomatic patient (II-3) in Family 2 and the younger member of Family 2 carried the mutation without the presence of an additional “map-dot-fingerprint” pattern, as previously described [16,22,23]. Therefore, not all individuals show this association, probably due to the incomplete penetrance of the mutations, typical of this corneal dystrophy. Coexistence of MECD and EBMD was described in a recent publication of Sneyers et al. reporting images of a 63-year-old man without genetic analysis [24]. Molecular genetic analysis of the present families confirmed this comorbidity.

According to the 3rd edition of the International Classification of Corneal Dystrophies (IC3D), MECD is classified as a “Category 1” with well-defined loci (12q13 for *KRT3* and 17q12 for *KRT12*), while EBMD is classified into “Category 3” and does not have a defined genetic locus, and most cases have no inheritance documented [23]. EBMD is usually considered to be degenerative and not hereditary or secondary to trauma; it is commonly present in adult life and rarely described in children. Only one publication identified a Transforming growth factor-beta-induced mutation in 5q31 [25]. Thus, in the present cases, EBMD, with the presence of fingerprint lines and intraepithelial dot opacities, is most likely to be degenerative and coexisting with MECD, which developed excessive cellular proliferation and inflammation, leading to basement membrane degradation.

Intraepithelial microcysts in the proband of Family 1 (II-2) were larger than reported [26,27,28]. ET-map showed irregularity with superior thinning, which may have been induced by the mechanical friction of blinking.

In Family 2, multimodal images and epithelial thickness mapping (ETM) provided relevant descriptive phenotypic information on the alterations, which appear to show more severe changes as the affected patients age. The microcyst dimensions agree with the findings of Hernández-Quintela et al. (1998) [27] but are smaller than the sizes described by other authors [26,28].

The ET-map obtained via AS-OCT demonstrated that patients carrying the *KRT3* nucleotide variant had a thicker central corneal epithelium (mean: 70.33 ± 7.57 µm OD; 70.33 ± 10.41 µm OS) compared to unaffected members and to normal values (approx. 53.7 ± 4.0 µm) reported by Abusamak (2022) [29]. As reported by Buffault J. et al. (2020) [22], the OCT pachymetry map demonstrated that patients with EBMD had a thicker corneal epithelium in the central and inferior regions. In our study, a correlation between epithelial thickness and microcyst density was observed in Family 2: patient II-1 had the thickest epithelium (79–82 µm) and the highest microcyst density (95–101 cysts/mm^2^) compared to patients I-2 and II-3 (epithelium 62–65 µm; density 25–73 cysts/mm^2^). Interestingly, the unaffected patient (II-2) showed a thinner ET-map (45–48 µm), possibly associated with dry eye and an increased compensatory blinking mechanism [22].

Regarding the limitations of this study, for Family 1, to detect the modifying effect on the severity of the pathology, it would be advisable to perform a co-segregation study of the *KRT3* c.250C>T (p.Arg84Trp) variant.

For both families, longer follow-up will be advisable, above all for monitoring the youngest patients over time to document the possible deterioration of corneal conditions and assess the effect of lubricants on disease progression.

In conclusion, this study expands the genetic spectrum of MECD by identifying a novel, likely pathogenic *KRT12* variant (c.1181T>C) (according to ACMG) as the primary molecular cause of the disease and by reporting, for the first time, its co-occurrence with a *KRT3* variant (KRT3 c.250C>T, p.Arg84Trp). The homozygous state of the *KRT12* variant in the proband, likely resulting from parental consanguinity, supports a gene–dosage effect that may contribute to the severity or complexity of the phenotype, whereas the *KRT3* variant may act as a genetic modifier.

We also highlight the association of MECD with epithelial basement membrane alterations in both families. The detailed characterization using IVCM and AS-OCT ET-Map provides a deeper understanding of the morphological changes and phenotypic variability in MECD, confirming the utility of this multimodal imaging approach for diagnosis and management.

## 4. Materials and Methods

This prospective study was approved by the Ethics Committee of the Centro de Oftalmología Barraquer (ethics number: 162_ MESSMANN2). Written informed consent was obtained from all participants before enrolment, following the tenets of the Declaration of Helsinki.

### 4.1. Clinical Evaluation

A total of 10 patients from two families were included: a two-generation Lebanese family (*n* = 4) (Family 1) and a three-generation Spanish family (*n* = 6) (Family 2) with clinically diagnosed MECD.

Clinical diagnosis was performed using standard ophthalmic evaluation performed at Centro de Oftalmología Barraquer, including best-corrected visual acuity, biomicroscopy, and fundoscopy. A Schirmer I test (normal values ≥ 10 mm in 5 min), NIBUT (Oftaltech, CSO, Florence, Italy; dry eye defined as ≤10 s), and corneal esthesiometry using the Cochet-Bonnet esthesiometer (Luneau Ophtalmologie, Chartres Cedex, France; maximum corneal sensitivity: 60 mm) were used to analyze the stability of the tear film and corneal sensitivity, which are affected in cases of ocular surface irregularities.

### 4.2. In Vivo Laser Confocal Microscopy (IVCM)

Corneal microstructure was assessed using IVCM HRT3 with a Rostock cornea module of the Heidelberg Retina Tomograph (RCM-Heidelberg Engineering GmbH, Heidelberg, Germany) and analyzed according to the procedure previously described [30]. IVCM was only performed in adult affected individuals.

### 4.3. Anterior Segment Optical Coherence Tomography (AS-OCT)

A spectral-domain OCT (Cirrus 5000 HD, Zeiss, Dublin, CA, USA) with anterior segment corneal adaptor lens was used to obtain corneal thickness and ET-map. The automated average measures were provided for 3 concentric ring-shaped zones on the cornea (central cornea: 0–2 mm; paracentral cornea: 2–5 mm; and mid-peripheral cornea: 5–7 mm), and ET-map was provided for 4 concentric zones (central: 0–2 mm; paracentral: 2–5 mm; mid-peripheral: 5–7 mm; and peripheral: 7–9 mm).

### 4.4. Genetic Analysis

Genomic DNA was extracted from peripheral blood of patients and available relatives using the QIAamp DNA Blood Maxi Kit (Qiagen, Hilden, Germany) and the prepIT•L2P (PT-L2P) reagent (DNA Genotek, Ottawa, ON, Canada).

DNA from the probands of Family 1 (II-2) and Family 2 (I-2) underwent NGS on the Illumina HiSeq platform utilizing a custom-designed targeted gene panel comprising 133 genes associated with anterior segment dysgenesis and dystrophies.

Sequencing reads were mapped to the human reference genome (hg19) using the GEM toolkit, allowing for up to four mismatches. Only properly paired, uniquely mapped reads without duplicates were retained. Alignment files were processed with Picard for read group addition and duplicate removal. Local realignment was performed using the Genome Analysis Toolkit (GATK), and variant calling was carried out using SAMtools. Functional annotation was performed using SnpEff with the GRCh37. Variants were further annotated with SnpSift using the following resources: dbSNP (build 137), population frequencies from the 1000 Genomes Project, the Exome Variant Server, and the Genome Aggregation Database (gnomAD). The predicted molecular and phenotypic effects of variants in anterior segment associated genes were assessed in silico based on conservation metrics and deleteriousness scores from dbSNP, including the Likelihood Ratio Test (LRT), Combined Annotation-Dependent Depletion, MutationTaster, PolyPhen-2, and Sorting Intolerant From Tolerant (SIFT). Candidate variants (classified as pathogenic, likely pathogenic, or of uncertain significance) were validated by Sanger sequencing and subjected to co-segregation analysis in available family members. All variants were classified according to the ACMG guidelines [31].

## Figures and Tables

**Table 1:** Demographic and clinical features and genetic results (Families 1 and 2).

Family Number	Age/Gender	Age at Dx	BCVA OD/OS	IOP OD/OS	Associated Disease	Symptoms	Slit-Lamp Examination
1 (I-1)	62/M	62	0.10/0.00	11/11	Pterygium (OD) cataract (OD)	Asymptomatic	Normal
1 (I-2)	52/F	52	−0.04/−0.04	13/13	Conjunctival melanosis	Asymptomatic	Epithelial microcyst
1 (II-1)	35/M	35	−0.08/−0.08	14/14	Papillomatous lesion on the lower eyelid	Asymptomatic	Normal
1 (II-2) *	30/M	28	0.00/−0.06	14/14	Seborrheic blepharitis Astigmatism Myopia	FBS, photophobia Temporary episodes of blurred vision Ocular pain Lacrimation	Epithelial microcysts Map-dot-like lesions Superficial punctate keratitis Subepithelial corneal scars
2 (I-2) *	72/F	36	0.26/0.26		None	FBS, RCE	Epithelial microcysts Map-dot-like lesions Subepithelial scars
2 (II-1)	50/M	14	−0.08/0.19	19/19	Amblyopia OS	FBS, photophobia CL intolerance	Epithelial microcysts Map-dot-like lesions Nodular lesion Subepithelial scars
2 (II-2)	48/M	NA	−0.0/−0.08		None	NA	Normal
2 (II-3)	44/F	7	−0.06/−0.04		None	Asymptomatic	Epithelial microcysts
2 (III-1)	15/F	NA	−0.11/−0.11		None	NA	Normal
2 (III-2)	7/M	7	0.10/0.10		Allergic conjunctivitis	Asymptomatic	Diffuse epithelial punctate lesions No microcysts

BCVA: best-corrected visual acuity measured with ETDRS (LogMAR); CL: contact lens; F: female; FBS: foreign body sensation; M: male; NA: not affected; OD: oculus dexter; OS: oculus sinister; RCE: recurrent corneal erosion; *: proband.

**Table 2:** IVCM findings in MECD-affected patients (Family 1 and 2).

Family Number	Microcyst Density (Cell/mm^2^ ± SD)	Microcyst Diameter (µm) (Max–Min)	Hyperreflective Material Density (Debris/mm^2^ ± SD)	Subepithelial Nerve Abnormalities	Bowman’s Layer Atrophy	Active Keratocytes in Stroma
1(II-2) *	98 ± 4 /106 ± 9	45.22–15.6/55.3–17.7	226 ± 16/212 ± 16	+	+	+
2 (I-2) *	25 ± 2/34 ± 4	58.3–15.6/31.1–13.6	261 ± 14/213 ± 10	+	+	+
2 (II-1)	95 ± 8/101 ± 8	55.4–14.7/52.2–17.8	89 ± 8/132 ± 9	+	+	+
2 (II-3)	52 ± 5/73 ± 7	40.3–13.0 /37.5–11.6	100 ± 9/134 ± 8	+	-	-

(*) proband; (+): presence; (-): absence. Some data present OD/OS.

## Data Availability

The original contributions presented in this study are included in the article. Further inquiries can be directed to the corresponding author.

## Abbreviations

ACMG	American College of Medical Genetics and Genomics
AS-OCT	Anterior segment optical coherence tomography
EBMD	Epithelial basement membrane dystrophy
ET-map	Epithelial thickness mapping
GATK	Genome Analysis Toolkit
IC3D	International Classification of Corneal Dystrophies
IVCM	In vivo confocal microscopy
LRT	Likelihood Ratio Test
MECD	Meesmann epithelial corneal dystrophy
NGS	Next-Generation Sequencing
NIBUT	Non-invasive tear breakup time
OD	Right eye
OS	Left eye
PT-L2P	prepIT•L2P
SIFT	Scale-invariant feature transform
VA	Visual acuity
